# Correction: *Amblyomma maculatum* Feeding Augments *Rickettsia parkeri* Infection in a Rhesus Macaque Model: A Pilot Study

**DOI:** 10.1371/journal.pone.0137598

**Published:** 2015-08-31

**Authors:** Kaikhushroo H. Banajee, Monica E. Embers, Ingeborg M. Langohr, Lara A. Doyle, Nicole R. Hasenkampf, Kevin R. Macaluso

## Notice of Republication

This article was republished on August 20, 2015, because the legend for Table 2 was erroneously typeset as part of the body text of the article. Please download this article again to view the correct version. The originally published, uncorrected article and the republished, corrected article are provided here for reference.

## Supporting Information

S1 File Originally published, uncorrected article. (PDF)

S2 File Republished, corrected article. (PDF)
