# Supplementary material for: Correction: Amblyomma maculatum Feeding Augments Rickettsia parkeri Infection in a Rhesus Macaque Model: A Pilot Study
Source: PLoS One. 2015 Aug 31;10(8):e0137598. doi: 10.1371/journal.pone.0137598 (PMC4554988; doi:10.1371/journal.pone.0137598)
Supplement: S2 File — (PDF) [file pone.0137598.s002.pdf]

RESEARCH ARTICLE

Amblyomma maculatum Feeding Augments *Rickettsia parkeri* Infection in a Rhesus Macaque Model: A Pilot Study

Kaikhushroo H. Banajee[1], Monica E. Embers[2], Ingeborg M. Langohr[3], Lara A. Doyle[4], Nicole R. Hasenkampf[2], Kevin R. Macaluso[1]*

1 Vector-borne Disease Laboratories, Department of Pathobiological Sciences, School of Veterinary Medicine, Louisiana State University, Baton Rouge, Louisiana, 70803, United States of America, **2** Division of Bacteriology and Parasitology, Tulane National Primate Research Center, Tulane University Health Sciences, Covington, Louisiana, United States of America, **3** Department of Pathobiological Sciences, School of Veterinary Medicine, Louisiana State University, Baton Rouge, Louisiana, United States of America, **4** Division of Veterinary Medicine, Tulane National Primate Research Center, Tulane University Health Sciences Center, Covington, Louisiana, United States of America

* kmacaluso@vetmed.lsu.edu

CrossMark
click for updates

OPEN ACCESS

Citation: Banajee KH, Embers ME, Langohr IM, Doyle LA, Hasenkampf NR, Macaluso KR (2015) *Amblyomma maculatum* Feeding Augments *Rickettsia parkeri* Infection in a Rhesus Macaque Model: A Pilot Study. PLoS ONE 10(8): e0135175. doi:10.1371/journal.pone.0135175

Editor: Ulrike Gertrud Munderloh, University of Minnesota, UNITED STATES

Received: May 16, 2015

Accepted: July 17, 2015

Published: August 5, 2015

Copyright: © 2015 Banajee et al. This is an open access article distributed under the terms of the Creative Commons Attribution License, which permits unrestricted use, distribution, and reproduction in any medium, provided the original author and source are credited.

Data Availability Statement: All relevant data are within the paper.

Funding: This work was supported by the National Institutes of Health (http://www.nih.gov/; grant numbers: 5P20GM103458, AI077784). KB was supported by T32 training grant# OD011124-08. The funders had no role in study design, data collection and analysis, decision to publish, or preparation of the manuscript.

Competing Interests: The authors have declared that no competing interests exist.

Abstract

Rickettsia parkeri is an emerging eschar-causing human pathogen in the spotted fever group of *Rickettsia* and is transmitted by the Gulf coast tick, *Amblyomma maculatum*. Tick saliva has been shown to alter both the cellular and humoral components of the innate and adaptive immune systems. However, the effect of this immunomodulation on *Rickettsia* transmission and pathology in an immunocompetent vertebrate host has not been fully examined. We hypothesize that, by modifying the host immune response, tick feeding enhances infection and pathology of pathogenic spotted fever group *Rickettsia* sp. In order to assess this interaction *in vivo*, a pilot study was conducted using five rhesus macaques that were divided into three groups. One group was intradermally inoculated with low passage *R*. *parkeri* (Portsmouth strain) alone (n = 2) and another group was inoculated during infestation by adult, *R*. *parkeri*-free *A*. *maculatum* (n = 2). The final macaque was infested with ticks alone (tick feeding control group). Blood, lymph node and skin biopsies were collected at several time points post-inoculation/infestation to assess pathology and quantify rickettsial DNA. As opposed to the tick-only animal, all *Rickettsia*-inoculated macaques developed inflammatory leukograms, elevated C-reactive protein concentrations, and elevated TH1 (interferon-γ, interleukin-15) and acute phase inflammatory cytokines (interleukin-6) post-inoculation, with greater neutrophilia and interleukin-6 concentrations in the tick plus *R*. *parkeri* group. While eschars formed at all *R*. *parkeri* inoculation sites, larger and slower healing eschars were observed in the tick feeding plus *R*. *parkeri* group. Furthermore, dissemination of *R*. *parkeri* to draining lymph nodes early in infection and increased persistence at the inoculation site were observed in the tick plus *R*. *parkeri* group. This study indicates that rhesus macaques can be used to model *R*. *parkeri* rickettsiosis, and suggests that immunomodulatory factors introduced during tick feeding may enhance the pathogenicity of spotted fever group *Rickettsia*.

Introduction

Within the past fifteen years, there has been a more than a four-fold increase in the number of tick-borne rickettsial disease cases in humans in the United States [1,2]. During this time frame, *Rickettsia parkeri*, a member of the spotted fever group (SFG) of *Rickettsia* transmitted by *Amblyomma maculatum* (the Gulf coast tick), was first identified as a human pathogen [3] with several cases reported in North and South America [4–10]. Clinical signs include fever, headache, malaise, myalgia, arthralgia, formation of a maculopapular rash and multiple eschars [4–10]. The eschar, along with milder symptoms, can be used to differentiate this disease from the more virulent *R. rickettsii*, which causes Rocky Mountain spotted fever (RMSF) [3,5]. An *R. parkeri*-associated eschar is a 0.5–2 cm in diameter, crusted, non-pruritic ulcer, surrounded by an indurated, erythematous halo. These lesions are characterized histologically by extensive necrosis of the epidermis and superficial dermis and prominent lymphohistiocytic vasculitis of dermal vessels [3,5,6,10].

Despite the recent emergence of *R. parkeri*, there have been few experimental models detailing the pathology, immune response and transmission of *R. parkeri* in mammalian hosts. A murine model has been developed in C3H/HeJ mice [11]. Using this model, *A. maculatum* nymph feeding subsequent to intradermal injection of *R. parkeri* resulted in increased pathogen load and associated pathology when compared to needle inoculation alone [12]. However, C3H/HeJ mice have a mutation in toll-like receptor 4 (TLR4) [13]. Signaling via TLR4 is needed for stimulation of dendritic cells and activation of natural killer cells, which kill SFG *Rickettsia*-infected cells [14]. Therefore, in order to study the immune response to *R. parkeri*, an immunocompentent host would be necessary. While immunocompentent cotton rats become infected with *R. parkeri* after subcutaneous injection, they do not develop characteristic eschars [15]. Eschars formed after intradermal inoculation of *R. parkeri* in a guinea pig model; however, the effect of inoculation on other organ systems and the underlying immunology were not evaluated [16]. In order to model human pathology and immune response, immunocompentent rhesus macaques were used in this pilot study.

As reviewed recently by Wikel [17], tick saliva contains substances that are capable of inhibiting a variety of cytokines, chemokines, and several other bioactive molecules. Tick saliva also has the ability to impair the function of several immune cells including natural killer cells, macrophages, neutrophils, and T and B lymphocytes [17]. While much of this work is based on other hard tick species, salivary molecules of *Amblyomma* sp. have been shown to inhibit chemokine, natural killer cell, and dendritic cell functions [18–22]. It is no surprise that with this immunosuppressive ability, tick feeding has been found to enhance transmission of a variety of tick-borne pathogens including viruses (Thogotovirus and tick-borne encephalitis virus) and bacteria (*Borrelia afzelii*, *B. burgdorferi*, *B. lusitaniae*, *Anaplasma marginale*, *A. phagocytophilum*, and *Francisella tularensis*) [17,23]. However, the effect of *A. maculatum* feeding on *R. parkeri* rickettsiosis and the immune response in a mammalian model has not been comprehensively studied.

The experiments detailed in this report were designed to reproduce disease caused by *R. parkeri* via intradermal inoculation during adult *A. maculatum* feeding in rhesus macaques as compared to two other treatments: *R. parkeri* inoculation and *A. maculatum* feeding alone. The broad hypothesis is that by modulating the host immune response, tick feeding enhances infection and pathology of pathogenic SFG *Rickettsia*. We demonstrated that tick feeding during *R. parkeri* inoculation resulted in larger areas of necrosis with delayed healing as compared to *R. parkeri* inoculation alone. Furthermore, greater neutrophilia and interleukin (IL)-6 concentrations were noted in animals inoculated during tick feeding. Lastly, in a tick + *R. parkeri* animal, rickettsial DNA was detected in a draining lymph node in the acute phase of infection

and in the skin at the inoculation site in the chronic phase of infection suggesting the possibility of greater dissemination and persistence of *Rickettsia* in response to tick feeding. Taken together, these results reveal the utility of a primate model of *R. parkeri* infection and demonstrate that tick feeding can modify the pathogenesis of tick-borne rickettsiosis.

Materials and Methods

Tick and *Rickettsia* Preparation

A colony of *R. parkeri*-free *A. maculatum* was maintained on rodents as previously described [12,24]. All animals that were used for tick-rearing purposes were housed at the Louisiana State University (LSU) Division of Laboratory Animal Medicine (DLAM) vivarium on a 12-hour light-dark cycle with ad libitum rodent feed and water. Animals were housed in social pairs or groups appropriate to the species until tick placement; at which point, they were housed individually in order to prevent partner manipulation of tick containment devices. Larvae were fed on adult BALB/c mice (LSU DLAM, Baton Rouge, LA, USA) that were housed on wire grates over fresh water, and engorged larvae were collected twice daily as the water was changed. Nymphal and adult ticks were fed on adult Sprague-Dawley rats (LSU DLAM) or adult Hartley guinea pigs (Charles River Laboratories, Wilmington, MA, USA) within capsules fashioned from plastic 50 ml conical tubes and attached with a 3:1 tree rosin to bee wax mixture. After tick collection following feeding to repletion and dropping off of their hosts, animals were humanely euthanized with carbon-dioxide followed by cervical dislocation. Animal care and use for tick rearing purposes was approved by the Louisiana State University Institutional Animal Care and Use Committee (IACUC) (Protocol Number: 13–034).

The ticks used in this experiment were determined to be free of *R. parkeri* via DNA extraction and traditional semi-nested PCR using the 190.70p and 190.602n and 190.70p and 190.701 primer pairs for *Rickettsia ompA* as previously described [12,25–27]. Thirty female and fifteen male adult ticks were utilized in this study. Semi-purified rickettsiae were recovered from *R. parkeri* (Portsmouth strain) [3] passage 4 infected Vero cells (3 days post-inoculation) using the modified protocol of Weiss et al. [28] as previously described [29]. Rickettsiae were enumerated after staining with the LIVE/DEAD BacLight Bacterial Viability Kit (Molecular Probes, Carlsbad, CA, USA) in a Petroff–Hausser bacterial counting chamber (Hausser Scientific, Horsham, PA, USA) and examined with a Leica microscope (Leica Microsystems, Buffalo Grove, IL, USA) [30]. The rickettsiae were resuspended in sucrose-phosphate-glutamic acid buffer (SPG) [31] to obtain the desired inoculation dose of 1×10^7 live rickettsiae/200 μL, a dose that is at the high end of the range of total *R. parkeri* DNA found in wild-caught *Amblyomma* ticks [32] and is similar to the dose used in previous animal models of rickettsioses [11,12,33–37]. The same volume of uninfected Vero cell culture was prepared in SPG as above with the exception of bacterial inoculation and counting.

Non-human Primates

The five adult male Indian rhesus macaques (*Macaca mulatta*) used in the study were housed at the Tulane National Primate Research Center. Practices in the housing and care of nonhuman primates conformed to the regulations and standards of the Public Health Service Policy on Humane Care and Use of Laboratory Animals, and the Guide for the Care and Use of Laboratory Animals. The Tulane National Primate Research Center (TNPRC) is fully accredited by the Association for Assessment and Accreditation of Laboratory Animal Care International. The IACUC at the TNPRC approved all animal-related protocols specific to this study, including *R. parkeri* inoculation, tick infestation and sample collection from nonhuman primates (Protocol number: P0222) and all efforts were made to minimize animal suffering. All animals

received standard primate feed as well as fresh fruit and enrichment daily, and had continual access to water. Primates were housed in pairs within treatment groups prior to and after tick infestation. Single housing was required during tick infestation in order to prevent partner manipulation of jackets and tick containment devices. Single cages are 4.3ft^2 x 30”. Pairs were housed in larger cages, which at a minimum provide at least 4.3ft^2 x 30” per animal. Animals greater than 10 kg were allocated twice this amount of space. All animals received standard enrichment tailored to the species as dictated by the Animal Welfare Act and outlined in the TNPRC Policy on Environmental Enrichment (e.g., objects to manipulate in cage, varied food supplements, foraging and task-oriented feeding methods, interaction with caregivers and research staff). All animal procedures were overseen by TNPRC veterinarians and their staff and their welfare was monitored daily. Complete physical exams (including evaluation of the integumentary, musculoskeletal, lymphatic, gastrointestinal, cardiovascular, and respiratory systems) were performed, and rectal temperatures and weights were taken prior to each procedure. In order to alleviate animal suffering, the macaques were anesthetized for all procedures with 5–8 mg/kg Telazol intramuscularly (IM) followed by ketamine in small increments of 2–5 mg/kg IM as needed. In addition to this anesthetic protocol, all animals were pre-emptively given 0.01 mg/kg buprenorphine IM as additional analgesia for biopsies. None of the animals in this study demonstrated any deterioration in physical condition that required euthanasia during the experiment as determined by the standard TNPRC endpoint policy; therefore, the experimental endpoint for this study was 31–35 days post-*R*. *parkeri*/Vero cell lysate inoculation (31–35 dpi). At this point, the macaques were humanely euthanized at via administration of 5–8 mg/kg Telazol IM and 0.01 mg/kg buprenorphine IM followed by an overdose with 156 mg/kg sodium pentobarbital via intracardiac injection, a method that is consistent with the recommended guidelines of the American Veterinary Medical Association. Tulane University complies with NIH policy on animal welfare, the Animal Welfare Act, and all other applicable federal, state and local laws.

Tick Feeding and *Rickettsia parkeri* Inoculation

The macaques were split into three groups as outlined in Fig 1. Two animals each were placed in the *R*. *parkeri*-only and the tick + *R*. *parkeri* groups, and one was placed in the tick-only group. All animals were shaved and fitted with primate jackets (Lomir Biomedical, Inc., Notre-Dame-de-l’Île-Perrot, QC, Canada) one week prior to tick infestation to allow the primates to become acclimated to them. The tick exposure groups were infested with five male and ten female adult ticks using a tick containment device as previously described [38]. The number of ticks was chosen based on the fact that they could comfortably feed and engorge in the space allowed within the containment device. Male ticks were placed on the host and allowed to attach one day after applying the tick containment device, followed by female tick infestation two days later to stimulate the production of pheromones secreted during male feeding, such as the attraction-aggregation-attachment pheromone, which facilitate female tick attachment and feeding [39]. The tick feeding sites and containment devices were assessed, cleaned, and reinforced as needed at 3, 7, and 12 days post female tick infestation. All of the animals were inoculated intradermally 13 days after jacket placement (3 days after female tick infestation for the tick groups) with three 200 μL injections of either partially purified Vero cell lysate or *R*. *parkeri* at the tick feeding site for the tick groups or at a similar location on the cranial back for the *R*. *parkeri*-only group. Ticks, containment devices, and jackets were removed 12 days after female tick infestation.

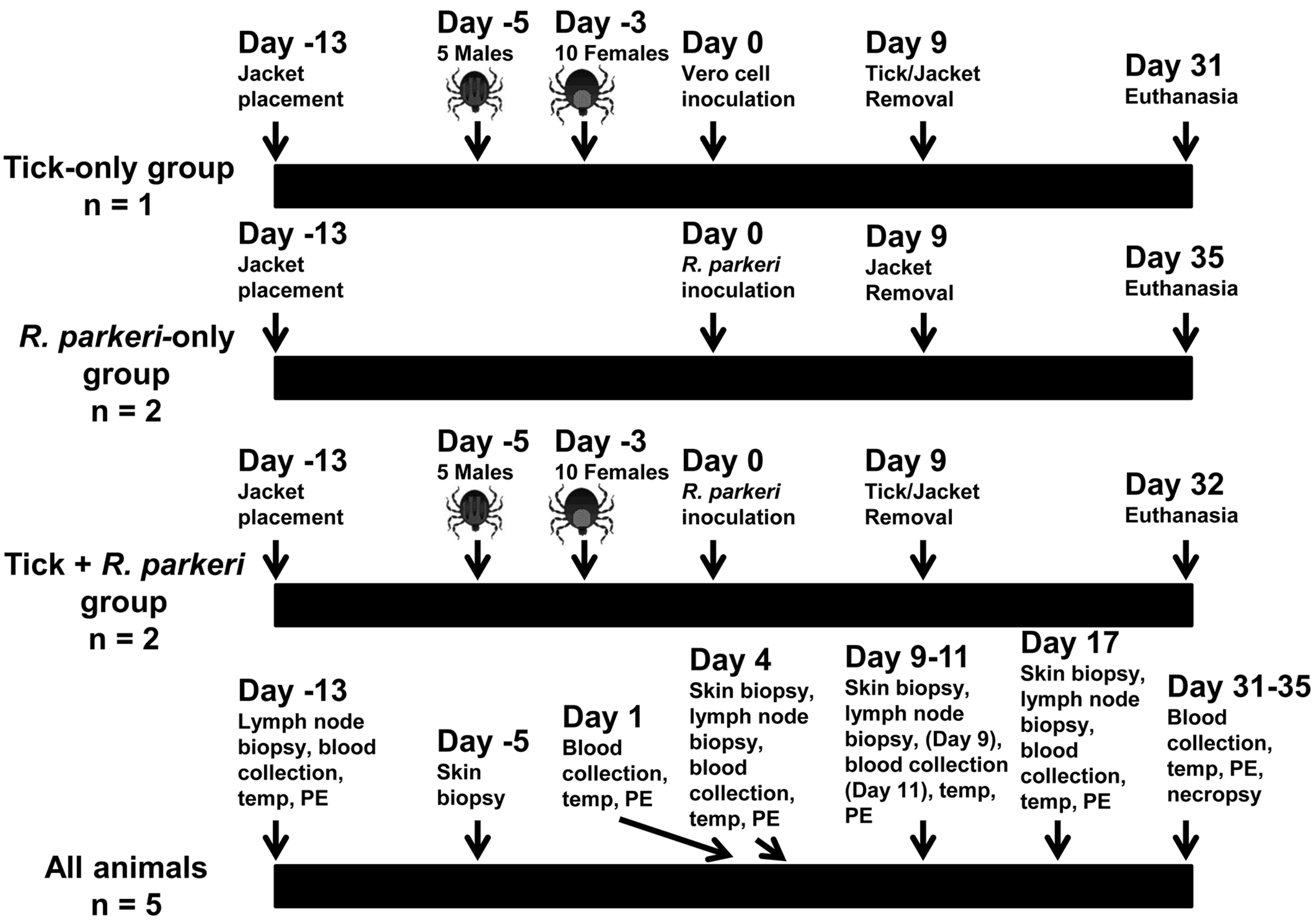

Fig 1. Experimental design for tick feeding, *R. parkeri*/Vero cell inoculation, and sample collection. Adult *Amblyomma maculatum* ticks were placed on the hosts as indicated. Either a partially purified low passage human isolate of *R. parkeri* or an uninfected Vero cell inoculum was administered at the indicated time points. Blood collection, physical exams (PE), rectal temperatures, and skin and lymph node biopsies were taken from all animals at the indicated time points. Complete necropsies were performed at the end of the study as indicated.

doi:10.1371/journal.pone.0135175.g001

Sample Collection

For all groups, blood, skin biopsies (4-mm punch), and excisional axillary or inguinal lymph node biopsies were collected at several time points as detailed in Fig 1. Skin biopsies were taken both at the site of *R. parkeri* inoculation/tick feeding and away from the inoculation/infestation site on the caudal dorsum. At necropsy, skin both at the inoculation site and at a distant location from the inoculation site, axillary and inguinal lymph nodes, lung, heart, liver, spleen, and bone marrow were collected. All tissues, including biopsies and tissues collected at necropsy, were split into two portions. One portion was frozen at -20°C until DNA extraction was performed and the other portion was fixed in Z-fix fixative (Anatech, Ltd., Battle Creek, MI, USA) and routinely processed for histopathological evaluation.

Hematology

Blood was collected into serum separator clot tubes for serum chemistry, cytokine concentrations, and indirect enzyme-linked immunosorbent assays (ELISAs) for anti-*R. parkeri* antibody determination. Serum chemistries including aspartate aminotransferase, alanine transaminase,

alkaline phosphatase, sodium, chloride, potassium, total protein, albumin, globulin, blood urea nitrogen, creatinine, glucose, and C-reactive protein were performed immediately. Serum for cytokine evaluation and ELISAs was separated from the cellular component after centrifugation and stored at -20°C. Blood was also collected into EDTA tubes for complete blood count (CBC) determination and DNA extraction for *R. parkeri* quantification. CBCs were performed immediately, whereas blood for DNA extraction was stored at -20°C. As part of the CBC, fresh blood smears stained with Diff-Quick (Siemens Corporation, Washington, D.C., USA) were evaluated in a randomized manner by a board-certified veterinary clinical pathologist to determine the manual leukocyte cell differential and to evaluate erythrocyte, leukocyte, and platelet morphology.

Serum cytokine concentrations of 23 analytes (granulocyte-colony stimulating factor [G-CSF], granulocyte macrophage-colony stimulating factor (GM-CSF), interferon [IFN]-γ, IL-10, IL-12/23 (p40), IL-13, IL-15, IL-17, IL-18, IL-1 receptor antagonist [IL-1ra], IL-1β, IL-2, IL-4, IL-5, IL-6, IL-8, monocyte chemotactic protein-1 [MCP-1], macrophage inflammatory protein-1α [MIP-1α], MIP-1β, transforming growth factor-α [TGF-α], tumor necrosis factor-α [TNF-α], vascular endothelial growth factor [VEGF], soluble cluster of differentiation 40 ligand [sCD40L]) were measured with a 23 plex Milliplex MAP non-human primate cytokine magnetic bead panel (EMD Millipore, Billerica, MA, USA) according to the manufacturer's instructions. Each sample was evaluated in duplicate without dilution, along with duplicates of seven dilutions of provided standards and a low and high concentration quality control sample provided by the manufacturer. Data were acquired on a Luminex 100 system and analyzed using bioplex manager software (Bio-Rad Laboratories, Hercules, CA, USA).

Indirect ELISAs to detect anti-*R. parkeri* IgG were performed on the serum samples from three time points (7 days prior to *R. parkeri* exposure, 11 dpi, and 31–35 dpi) as adapted from a previously described protocol [40]. Briefly, half of the wells of 96-well plate were coated with *R. parkeri* whole cell antigen and half without antigen followed by incubation overnight at 4°C with blocking buffer (5% skim milk/0.1% Tween-20 in phosphate-buffered saline). The macaque serum samples were used as primary antibody, goat anti-monkey IgG conjugated to horseradish peroxidase (Kirkegaard & Perry Laboratories, Inc., Gaithersburg, MD, USA) diluted 1:5000 in blocking buffer was used as the secondary antibody, and the reaction was visualized with the OptEIA tetramethylbenzidine substrate reagent set (BD Biosciences, San Jose, CA, USA). After a 15-minute incubation, the reaction was stopped with 2N sulfuric acid, and optical densities (ODs) were read with a Spectramax M2 spectrophotometer (Molecular Devices, Sunnyvale, CA, USA) at 450 nm minus the absorbance at 650 nm. Additionally, serum from a mouse previously inoculated with *R. parkeri* followed by goat anti-mouse IgG conjugated to horseradish peroxidase (Thermo Fisher Scientific, Waltham, MA, USA) as the secondary antibody and wells without serum were used as positive and negative controls, respectively. Samples were run in triplicate and the mean ODs were calculated after subtracting the ODs in the wells without antigen from the ODs in the wells with antigen. Samples that were positive at 1:64 were then subjected to two-fold serial dilutions until negative to get an endpoint titer, as has been previously reported [5]. A sample was considered positive at a certain dilution if the mean of the net ODs was greater than 0.200 or greater than the mean OD of the negative controls plus three standard deviations, whichever was larger. Endpoint titers were determined to be the highest positive dilution for each sample [40].

Histopathology and Immunohistochemistry

After fixation, tissues were routinely embedded in paraffin, sectioned, and stained with hematoxylin and eosin (H&E) for histopathological evaluation. Tissue sections were evaluated in a

randomized, blinded manner by a board-certified veterinary anatomic pathologist. Skin from the inoculation sites and lymph node sections for all groups were assessed by immunohistochemistry (IHC) for the presence of *Rickettsia* using an anti-RC_{PFA} polyclonal rabbit primary antibody [41]. Cross-reactivity of this antibody to *R. parkeri* was confirmed by staining *R. parkeri* (Portsmouth strain) infected Vero cells. Briefly, slides were stained using a DAKO autostainer LINK 48 after proteinase K antigen retrieval (Dako, Carpinteria, CA, USA) with anti-RC_{PFA} (1:2000) and a biotinylated anti-rabbit secondary antibody (Vector Laboratories, Burlingame, CA, USA), and visualized using the avidin/biotinylated enzyme complex (Vector Labs) and the ImmPACT NovaRED peroxidase substrate (Vector Labs), followed by counterstaining with Mayer's hematoxylin. False positives due to non-specific binding of the secondary antibody were ruled out by comparing sample staining to staining in tissue sections that were stained without primary antibody.

PCR for Detection of Rickettsial DNA

Genomic DNA was extracted from blood and tissue samples using the DNeasy Blood and Tissue Kit (Qiagen, Germantown, MD) according to the manufacturer's instructions. Extracted DNA was stored at -80°C until real-time quantitative PCR (qPCR) was performed. In order to detect rickettsial and rhesus macaque DNA, *Rickettsia ompB* primers [42], an *R. parkeri* species-specific fluorescent-labeled probe (5'-/Cy-5/TTTG+A+G+C+A+G+CA/3IABkFQ/-3'), and rhesus macaque oncostatin M (*OSM*) primers and probe [43] were used. The *Rickettsia ompB* gene is a single copy gene that encodes a common rickettsial surface antigen protein, and the rhesus macaque *OSM* gene is a single copy gene that encodes the oncostatin M cytokine. To quantify *R. parkeri* DNA in macaque tissues, serial dilutions of a plasmid containing single-copies of the *R. parkeri ompB* and rhesus macaque *OSM* genes were amplified along with the unknown samples, environmental DNA extraction controls, and water (negative controls) using iTaq Universal Probes Supermix (Bio-Rad Laboratories) and the LightCycler 480 system II (Roche, Indianapolis, IN, USA) as previously described [44]. To confirm that the positive qPCR results were due to *R. parkeri* and to assess potential transmission of *Candidatus* "R. andeanae" (an *A. maculatum* symbiont), a 631bp segment of the *Rickettsia ompA* gene was amplified from all qPCR positive tissue sample DNA extracts and skin DNA extracts at the site of tick infestation at 4 and 9 dpi in the tick-only animal using 190.70p and 190.701 primers and thermocycling conditions as previously described [25–27]. The products were visualized on a 2% agarose gel. Amplicons were extracted from the gel using a PCR Clean-up System (Promega, Madison, WI, USA), cloned into pCR 4-TOPO vector and at least five clones from each sample were sequenced at Louisiana State University. Nucleotide similarities of the sequences were evaluated on the GenBank BLAST database (http://blast.ncbi.nlm.nih.gov/Blast.cgi). Engorged female ticks from the animals in this experiment were allowed to oviposit in humidified chambers and eggs from these ticks were allowed to hatch. Genomic DNA was extracted from pools of 10–20 larvae as described above after freezing in liquid nitrogen and grinding them with a sterile pestle. Traditional PCR with the 190.70p and 190.701 primers, cloning, and sequencing were performed on the amplicons as described above.

Results

Tick Feeding

At 3 days post female tick placement, the majority of the female ticks had attached in all of the tick infestation groups (7/10 in the tick-only group, 9/10 and 10/10 in the tick feeding + *R. parkeri* animals). Furthermore, all males were attached at this time, except for one in the tick-only group. The remaining ticks were stuck in the glue surrounding the tick containment apparatus

and did not feed. At the time of tick removal, most of the females that had attached were fully engorged in all tick infestation groups.

Clinical data and Hematology

No differences in weight or temperature were noted between treatment groups during the study. Mild to marked peripheral lymphadenopathy was noted in all animals from 4 dpi to 11 dpi primarily affecting the axillary lymph nodes. At 1 dpi, moderate neutrophilia (greater than 4-fold pre-inoculation values) was noted in both primates in the tick + *R. parkeri* group as compared to mild neutrophilia (less than 3-fold baseline concentrations) in both *R. parkeri*-only primates (Fig 2A). All of these animals had mild neutrophilia at 4 dpi that resolved by the time of necropsy in all animals except for macaque #1 in the tick + *R. parkeri* group. The tick-only macaque developed mild neutrophilia at 4 dpi (less than 3-fold pre-inoculation levels), with values returning to baseline at necropsy. All of the animals inoculated with *R. parkeri* were lymphopenic at 1 and 4 dpi (less than or equal to half of baseline values), except for macaque #1 in the *R. parkeri*-only group, with values returning to baseline in all animals by the date of necropsy (Fig 2B). There were no apparent relevant differences between treatment groups for the rest of the CBC data.

C-reactive protein (CRP) concentration was mildly to markedly elevated (5 to greater than 50-fold increase from pre-inoculation concentrations) at 1 dpi in all *R. parkeri*-inoculated animals, with the highest concentration in primate #2 from the tick + *R. parkeri* group (Fig 2C). At 4 dpi, the CRP concentrations in these animals were mildly to moderately increased (5 to 18-fold pre-inoculation values), and returned to baseline for the remainder of the study. The tick-only macaque had mild elevation (less than 4-fold) in CRP concentration at 11 dpi only. There were no apparent relevant differences between treatment groups for the rest of the chemistry analytes evaluated during the study.

There were 17-20-fold increases in IL-6 concentrations in both of the tick + *R. parkeri* macaques at 1 dpi as compared to pre-inoculation values, with moderate elevations (8-12-fold baseline concentrations) noted at 4 dpi for the same two animals and macaque #1 from the *R. parkeri*-only group (Fig 3A). Moderate elevations (13-fold greater than pre-inoculation values) were noted in IFNγ concentration in primate #1 from the tick + *R. parkeri* group at 1 dpi with mild elevations (less than 7-fold pre-inoculation data) in all *R. parkeri*-inoculated animals at 4 dpi (Fig 3B). Also, there were mild increases (1.7 to 2.3-fold greater than baseline) in IL-15 concentration in both animals from the tick + *R. parkeri* group as well as *R. parkeri*-only macaque #2 at 4 dpi, with mild increases (1.5-fold greater than pre-inoculation data) at 4 dpi and 11 dpi in the tick-only animal. There were no apparent differences between groups for the remainder of the cytokines evaluated. All animals inoculated with *R. parkeri* had anti-*R. parkeri* IgG titers of at least 1:256 at 11 dpi with at least a 4-fold increase in titers by 31–35 dpi (Table 1). Anti-*Rickettsia* IgG was not detected in the tick-only animal during the experiment, nor in any of the animals prior to inoculation.

Gross Pathology

At 4 dpi, the skin at the site of tick infestation in the tick-only animal was diffusely erythematous, raised and thickened, encompassing the majority of the 5-cm tick containment area (Fig 4A). In the *R. parkeri*-inoculated animals, at 4 dpi, eschars formed at all inoculation sites (Fig 4B). In the *R. parkeri*-only group, these eschars were characterized by crusted ulcers that measured approximately 0.5–1 cm in diameter and were surrounded by 0.5–1.5 cm erythematous halos. The eschars were larger in both tick + *R. parkeri* primates, with areas of ulceration measuring up to 1.5 × 3 cm surrounded by diffusely erythematous, raised, and thickened skin

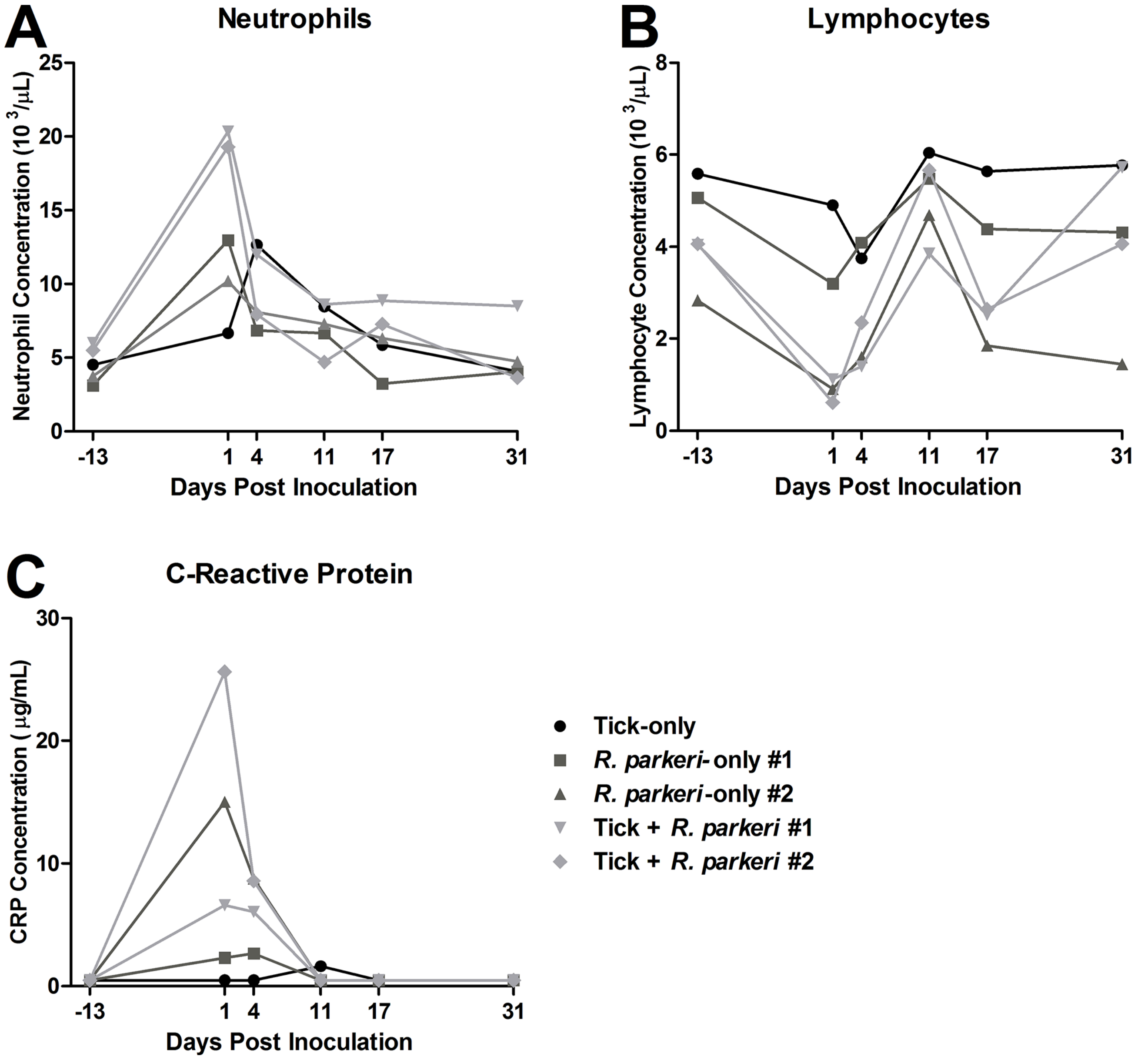

Fig 2. Evidence of an acute phase inflammatory response in response to *R. parkeri* inoculation. Comparisons of neutrophil (A), lymphocyte (B), and C-reactive protein (C) concentrations in peripheral blood of all animals at the various time points indicated. Neutrophilia, lymphopenia, and elevated C-reactive protein were noted in the acute phase of infection after *R. parkeri* inoculation with greater neutrophilia noted in the tick + *R. parkeri* group. For presentation purposes all of the final time points are plotted as 31 dpi as opposed to 31, 32, and 35 dpi for the tick-only, tick feeding + *R. parkeri*, and *R. parkeri*-only groups, respectively.

doi:10.1371/journal.pone.0135175.g002

of up to 5 cm in diameter (Fig 4C). At 9 dpi, the *R. parkeri*-only eschars began to heal with scar formation as opposed to increased erythema and ulceration that developed in all tick infestation groups. At necropsy, eschars in the *R. parkeri*-only primates had been replaced by scars measuring up to 0.1 × 0.3 cm (Fig 4D); whereas, healing ulcers with scar tissue were noted in all tick infestation groups that measured up to approximately 1 × 2 cm in the tick + *R. parkeri* macaques (Fig 4E). These healing ulcers were surrounded by maculopapular rashes measuring approximately 3–6 by 4.5–6 cm in both of the tick + *R. parkeri* macaques.

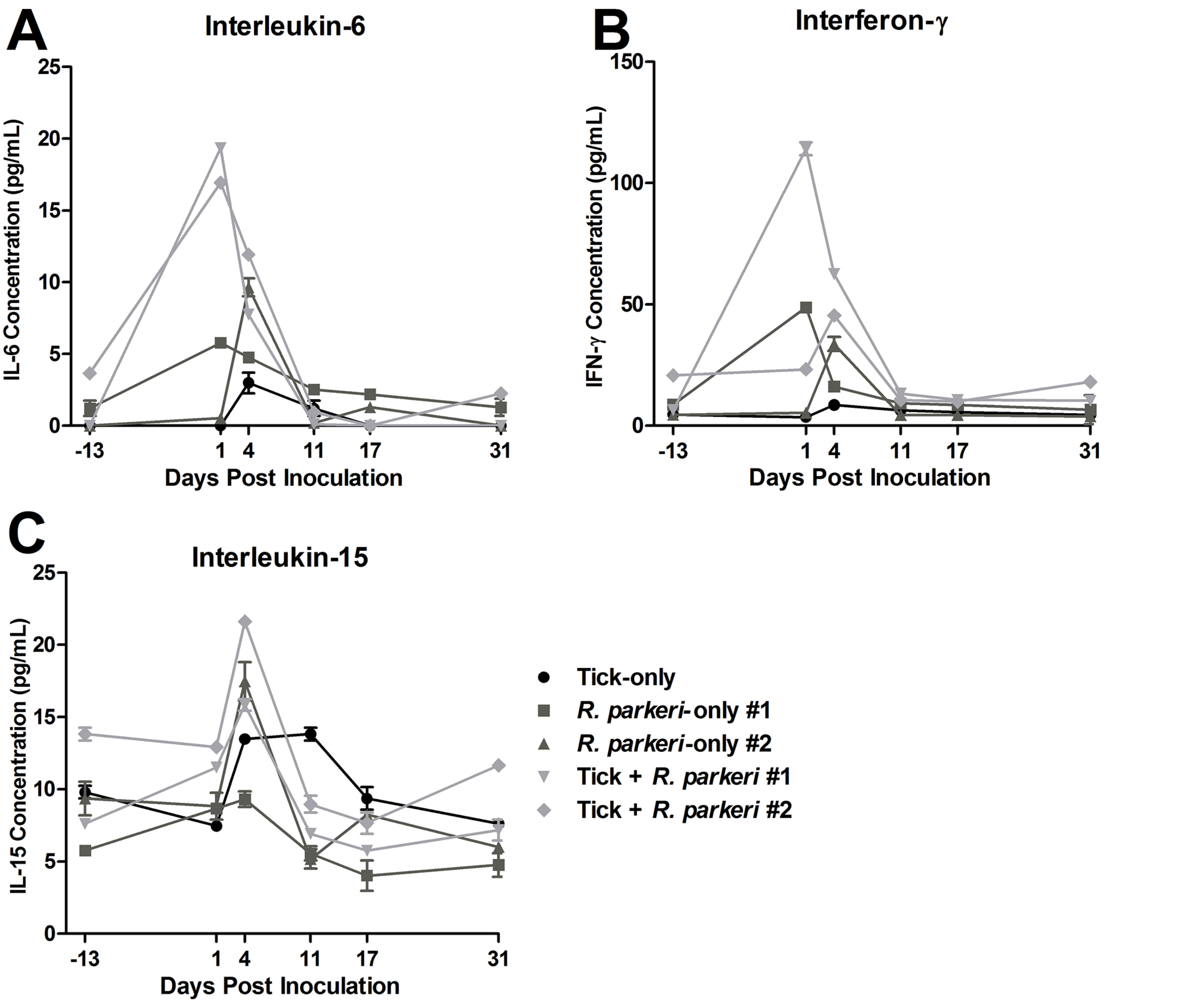

Fig 3. Concentrations of serum inflammatory cytokines are increased in response to *R. parkeri* inoculation. Comparisons of interleukin-6 (D), interferon γ (E), and interleukin-15 (F) concentrations in serum of all animals at the various indicated time points as determined by a magnetic cytokine bead panel kit. Measurements were performed in duplicate with the bars indicating standard error. For presentation purposes all of the final time points are plotted as 31 dpi as opposed to 31, 32, and 35 dpi for the tick-only, tick feeding + *R. parkeri*, and *R. parkeri*-only groups, respectively.

doi:10.1371/journal.pone.0135175.g003

Histopathology and Immunohistochemistry

The cutaneous histologic findings are summarized in Table 2. At 4 dpi, marked, diffuse dermatitis extending throughout the superficial and deep dermis and characterized by infiltration of many neutrophils and fewer macrophages was observed in all macaques, except the *R. parkeri*-only macaque #1, which had a moderate perivascular dermatitis consisting primarily of neutrophils with fewer macrophages. Epidermal necrosis was found only in the *R. parkeri*-inoculated animals (Fig 5A and 5B, Table 2). At 9 dpi, moderate to marked diffuse infiltration of the superficial and deep dermis by macrophages and neutrophils was noted in the tick + *R. parkeri* animals with moderate to marked epidermal necrosis, as opposed to mild perivascular

Table 1. Rise in anti-*R. parkeri* IgG titers in response to *R. parkeri* inoculation.

Animal	Pre-exposure (-7 dpi)	11 dpi	Necropsy (31–35 dpi)
Tick-only	-	-	-
R. parkeri-only #1	-	1:8,192	1:32,768
R. parkeri-only #2	-	1:256	1:4,096
Tick + *R. parkeri* #1	-	1:256	1:2,048
Tick + *R. parkeri* #2	-	1:256	1:4,096

All animals inoculated with *R. parkeri* had detectable anti-*R. parkeri* IgG during the acute phase of infection with at least a 4-fold increase in titers during convelscence as determined via an indirect ELISA.—designates that the samples are negative (titers <1:64)

doi:10.1371/journal.pone.0135175.t001

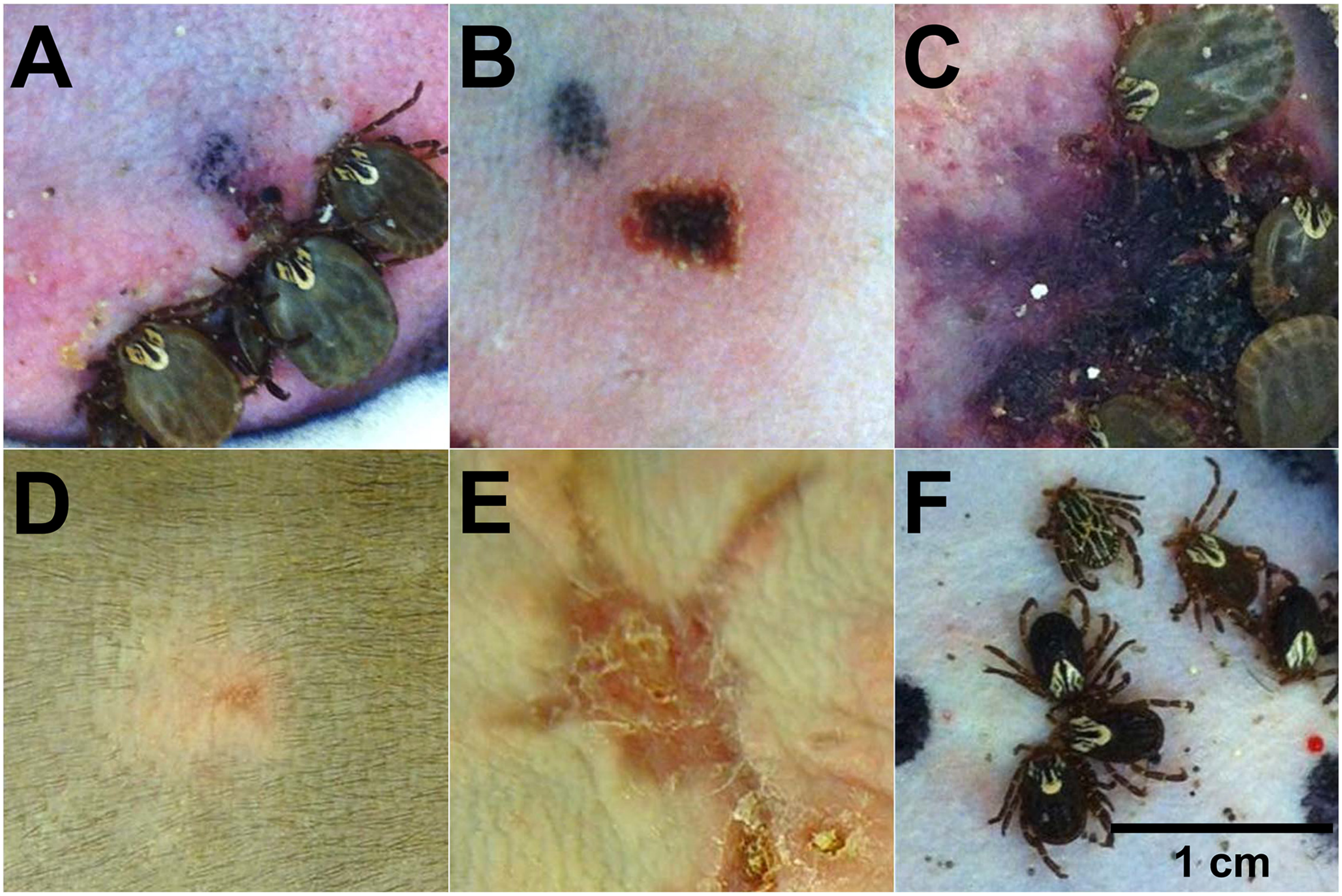

Fig 4. Eschars form after intradermal *R. parkeri* inoculation and are exacerbated by tick feeding during inoculation. Photographs of tick feeding/inoculation lesions of each group at 4 dpi (A-C), the same locations as pictured in B and C at 31–35 dpi (D and E), and another tick feeding/inoculation site at 0 dpi for comparison (F). (A) Tick feeding alone results in cutaneous erythema at 4 dpi. (B) Intradermal inoculation of *R. parkeri* results in eschar formation (well circumscribed ulcer surrounded by an erythematous halo) at 4 dpi. (C) Intradermal inoculation of *R. parkeri* during tick feeding results in a large area of necrosis surrounded by erythema at the inoculation site at 4 dpi. (D) *R. parkeri* inoculation alone results in the formation of a small scar at 35 dpi. (E) A large healing ulcer has replaced the eschar from the tick feeding + *R. parkeri* animal at 32 dpi. (F) No gross alterations are noted at the time of *R. parkeri* inoculation (3 days post female tick infestation) for comparison. Black marks were made adjacent to inoculation sites.

doi:10.1371/journal.pone.0135175.g004

Table 2. Marked dermatitis and epidermal necrosis in response to *R. parkeri* inoculation.

Animal	Epidermal Necrosis				Dermatitis				Anti-*Rickettsia* IHC			
	4 dpi	9 dpi	17 dpi	31–35 dpi	4 dpi	9 dpi	17 dpi	31–35 dpi	4 dpi	9 dpi	17 dpi	31–35 dpi
**Tick-only**	0	0	0	+	+++*	+	+	++	+	0	0	0
***R. parkeri*-only #1**	+++	+	0	0	++	+	0	+	++	++	0	0
***R. parkeri*-only #2**	+++	+	0	0	+++*	+	0	0	+++	+++	0	0
**Tick + *R. parkeri* #1**	+++	+++	++	0	+++*	+++*	+	++	++	++	0	0
**Tick + *R. parkeri* #2**	+++	++	+	0	+++*	++*	+	++	+	++	0	0

Histopathologic findings associated with intradermal inoculation of *R. parkeri* include marked epidermal necrosis and dermatitis during the acute phase of infection. Tick feeding during *R. parkeri* inoculation resulted in persistence of dermatitis and dermal vasculitis in the chronic phase of infection. Anti-*Rickettsia* immunohistochemistry revealed rare to many organisms in the skin at inoculation site during the acute phase of infection. 0 = absence of the specified parameter, + = mild histologic change (finding is rare to infrequent at high-power), ++ = moderate histologic change (change is found in multiple high-power fields or large foci are present in selected areas), +++ = marked histologic change (changes are frequently observed in multiple high-power fields or change is severe in focal areas). * Denotes diffuse dermatitis affecting the superficial and deep dermis as opposed to perivascular dermatitis denoted by the lack of an *.

doi:10.1371/journal.pone.0135175.t002

dermatitis characterized by aggregates of variable numbers of neutrophils, macrophages, lymphocytes and plasma cells noted in the tick-only and *R. parkeri*-only macaques with mild epidermal necrosis in the *R. parkeri*-only animals. At 17 dpi and at necropsy, mild to moderate perivascular lymphocytic to lymphoplasmacytic inflammation was noted in the tick infestation groups with mild to moderate epidermal necrosis noted in the tick + *R. parkeri* group at 17 dpi and mild epidermal necrosis in the tick-only group at 31 dpi. The *R. parkeri*-only group had no significant histopathological lesions at these time points except for the *R. parkeri*-only macaque #1, which had mild perivascular lymphoplasmacytic inflammation at necropsy. Furthermore, marked dermal vasculitis was noted in the *R. parkeri*-only macaque #2 at 4dpi with mild vasculitis noted in the tick + *R. parkeri* animal #1 at 4 and 9 dpi. This vasculitis was characterized by intramural fibrin deposition, endothelial cell necrosis/degeneration, and/or inflammatory cells (neutrophils and macrophages) within vessel walls (as depicted in Fig 5B). Mild to moderate lymphadenitis characterized by infiltrates of macrophages and neutrophils with lymphoid hyperplasia was noted in all animals at various time points after inoculation/infestation. No other significant lesions were noted in the other tissues collected. Immunohistochemical staining revealed few to many positively staining coccobacilli primarily within macrophages and fewer within neutrophils in both *R. parkeri*-inoculated groups at 4 and 9 dpi (Fig 6B and 6C, Table 2). Rare organisms were also noted in macrophages in tick infestation site in the tick-only animal at 4 dpi (Fig 6A, Table 2) and in a lymph node from the tick + *R. parkeri* macaque #2 at 4 dpi.

PCR for Detection of Rickettsial DNA

R. parkeri DNA was detected in the skin at the inoculation site in all of the *R. parkeri*-inoculated animals via qPCR at 4 and 9 dpi, with lower copy numbers detected in both tick + *R. parkeri* animals at 9 dpi (Fig 7). Furthermore, *R. parkeri* DNA was detected at the cutaneous inoculation site at necropsy and in a lymph node at 4 dpi from the tick + *R. parkeri* monkey #2. No rickettsial DNA was detected in the other tissue or blood samples from any animal via qPCR, including all tissues from the tick-only group at all time points, the extraction and negative control samples. qPCR positive tissue samples were then subjected to traditional PCR for sequencing of a segment of rickettsial *ompA*. Sequence analysis of amplicons from all of the

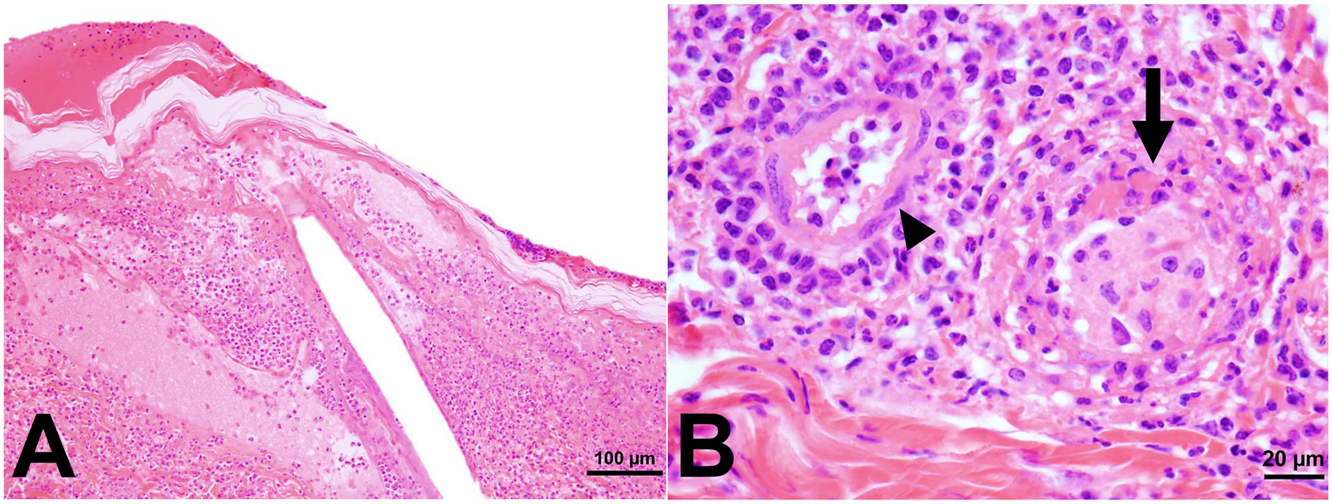

Fig 5. Intradermal inoculation of *R. parkeri* results in marked diffuse dermatitis characterized by infiltrates of neutrophils and macrophages, epidermal necrosis, and dermal vasculitis at 4 dpi. Photomicrographs of an H&E-stained skin section from a primate from the *R. parkeri*-only group at 4 dpi. (A) The epidermis is diffusely necrotic and superficial dermis is effaced by inflammatory cells. (B) Magnified view showing a dermal vessel (arrow) effaced by neutrophils and macrophages (vasculitis) and another dermal vessel with intact endothelium (arrowhead) surrounded by neutrophils and macrophages.

doi:10.1371/journal.pone.0135175.g005

qPCR positive tissue samples revealed a sequence identity of ≥ 99% with several different strains of *R. parkeri* (GenBank accession numbers CP00341.1, KF782320.1, U43802.1, FJ

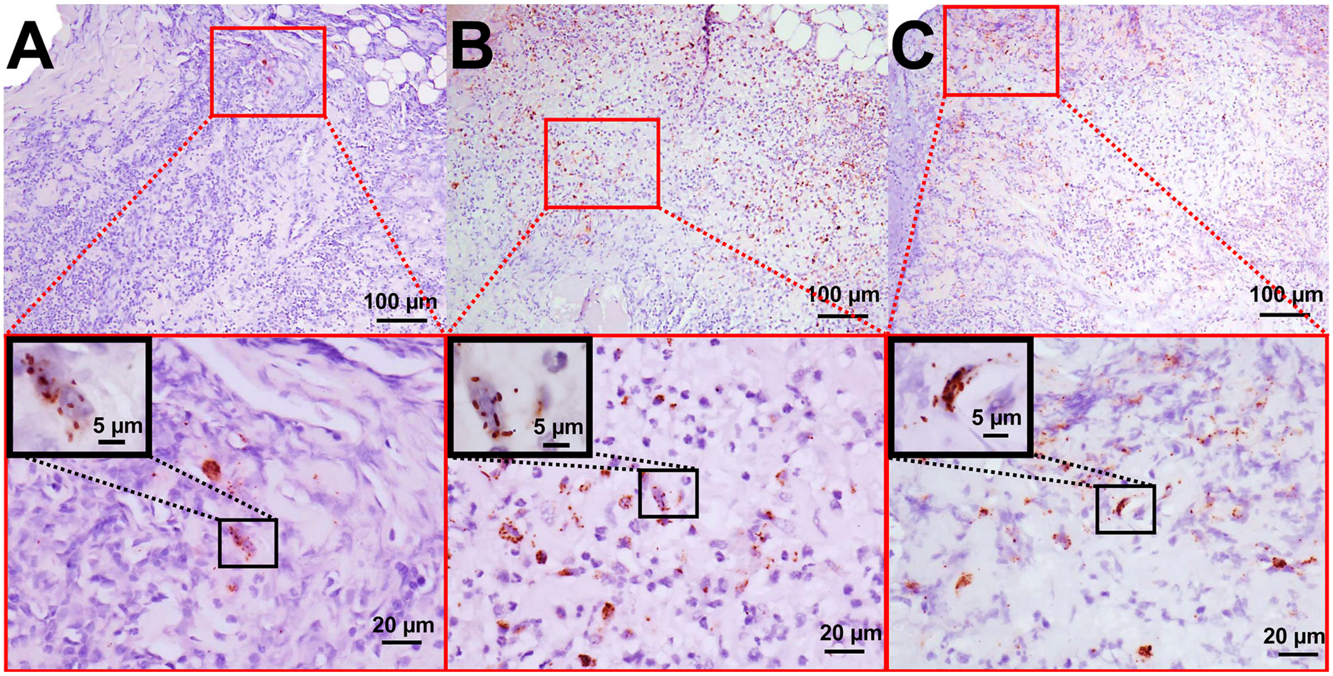

Fig 6. Anti-*Rickettsia* immunohistochemistry demonstrating numerous organisms in the skin of animals inoculated with *R. parkeri* at 4 dpi as opposed to rare *Rickettsia* in the tick-only animal. Photomicrographs of skin sections stained with a polyclonal anti-*Rickettsia* antibody at 4 dpi. (A) Rare cells contain positive, brown-staining, rickettsial organisms in the tick-only animal. (B) Abundant positive, brown-staining, organisms in a section from an animal in the *R. parkeri*-only group. (C) Similarly, many organisms are noted in an animal from the tick + *R. parkeri* group. The red-framed images at the bottom are higher magnification views of the red-boxed areas in the top images. Black-framed insets are higher magnification images of the black-boxed areas and highlight the coccobacilli morphology of the positively stained rickettsial organisms.

doi:10.1371/journal.pone.0135175.g006

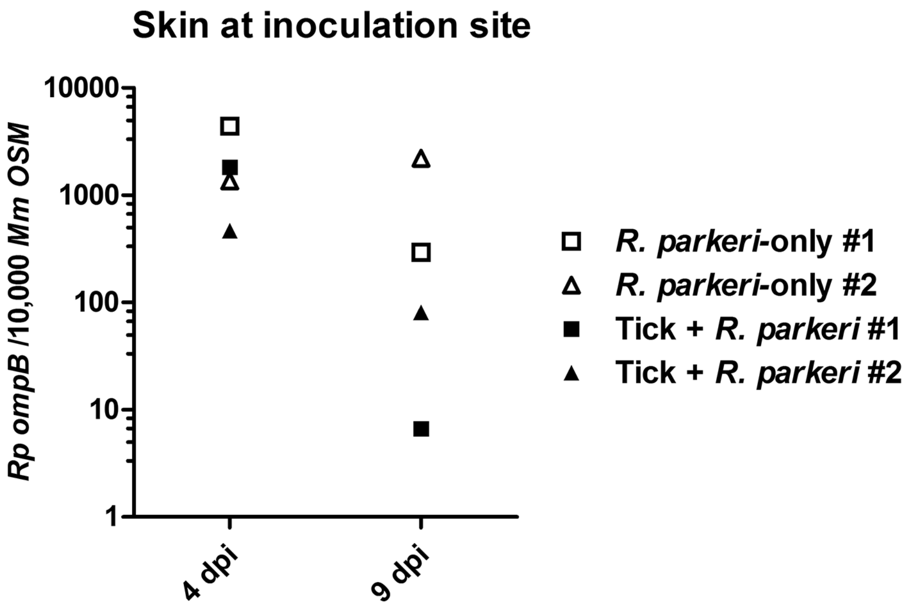

Fig 7. Rickettsial DNA was detected in the skin of *R. parkeri*-inoculated animals at 4 and 9 dpi. Rickettsial load as detected by qPCR in skin samples from 4 and 9 dpi expressed as *R. parkeri ompB* copies per 10,000 *M. mulatta OSM* copies. No rickettsial DNA was isolated from the tick-only macaque at any time point.

doi:10.1371/journal.pone.0135175.g007

986616.1, JX134641.1, KC003476.1, EU715288.1, and FJ172358.1). No amplicons were observed after traditional PCR using skin DNA extracts at the site of tick infestation from the tick-only animals at 4 and 9 dpi as template. Ten of 26 (38%) engorged female ticks collected in this experiment laid eggs that produced viable larvae. Rickettsial DNA was detected in one of the 10 larval pools (10% positive). This larval pool came from a female tick from the *R. parkeri* + tick macaque #2. Sequence analysis of this amplicon revealed a sequence identity of 100% to two strains of *Candidatus* "Rickettsia andeanae" (GenBank accession numbers KF179352.1 and KF030932.1).

Discussion

In this study, rhesus macaques were shown to be a suitable animal model of *R. parkeri* rickettsiosis, developing an acute phase inflammatory response, lymphadenopathy, anti-*R. parkeri* IgG, and characteristic eschars and maculopapular rashes with histologic evidence of dermal vasculitis after intradermal inoculation. The route of rickettsial inoculation used in this study, intradermal inoculation during tick feeding, while not replicating natural tick transmission of *R. parkeri*, was chosen in order to evaluate the effect of tick feeding on rickettsial pathogenesis as compared to the same dose of *R. parkeri* inoculated alone. If tick inoculation of *R. parkeri* was used instead, an appropriate *Rickettsia*-only control would be lacking as the dose and time-course of tick inoculation of *R. parkeri* remains undefined. Despite the fact that too few animals were utilized to perform statistical analysis, several conclusions can be made from this pilot study. All four rhesus macaques that were inoculated with *R. parkeri* developed an inflammatory leukogram characterized by mild to moderate neutrophilia and lymphopenia. Furthermore, moderate to marked elevations in CRP concentration, a major acute phase protein in rhesus macaques [45], and IL-6 concentration were noted during the same time frame. These abnormalities indicate activation of the innate immune response. Local inflammatory mediators, such as IL-6, are produced by innate immune cells in response to foreign substances [46],

in this case *R*. *parkeri*. This leads to production of acute phase proteins, like CRP, by hepatocytes [45,47] and release of neutrophils from the bone marrow storage pool within hours after the inciting stimulus [48]. Inflammatory mediators also cause reduction of the circulating lymphocyte pool due to multiple factors including increased migration to inflamed tissues, increased homing to lymph nodes, and decreased migration from lymphoid tissue back to blood [49]. A similar pattern of inflammation was noted in experimental *R*. *parkeri* infection in mice [11] as well as in natural infection of humans with *R*. *conorii* [50]. Elevation of serum IFNγ and IL-15 concentrations were also noted in *R*. *parkeri*-inoculated animals at 1 and 4 dpi indicating evidence of a TH1 response in these animals, which has been well described in SFG rickettsiosis [50,51]. Mild increases in serum IL-15 concentrations were also noted in the tick-only animal at 4 and 11 dpi, which is unexpected as tick feeding has been shown to downregulate the TH1 response [17,52–54]. While this finding could simply be an anomaly due to subject variability, further study is needed to define the role of this cytokine in the response to tick infestation. Tick feeding has also been shown to result in a TH2 response [17,52–56]; however, differences in TH2 cytokines were not detected in the serum of tick infested animals in this study, which could be attributed to the fact that these cytokines act locally at the feeding site and are not produced in large enough quantities to be detected in the peripheral blood. However, it is worth noting that many of the previous studies reporting cytokines induced by tick feeding were performed in BALB/c mice, which have a TH2-biased immune response [57–59]. Future experiments should include evaluation of cutaneous cytokine concentrations at the tick bite site in larger numbers of non-human primates to see if the Th1 versus Th2 cytokine paradigm is valid in this species. Furthermore, all animals inoculated with *R*. *parkeri* developed anti-*Rickettsia* IgG titers greater than or equal to 1:256 at 11 dpi with at least a 4-fold increase in convalescent titers indicating exposure and the appropriate antibody response to the pathogen [5]. Although rickettsial infections are typically associated with fever, elevated body temperature was not detected in any of the animals during the study. All animals were anesthetized during temperature evaluations; therefore, the induced hypothermia could have masked a potential fever. Continuous temperature monitoring could be of benefit to detect fever in future studies, as has been reported in rhesus macaques inoculated with *B*. *turicatae* [60].

Experimentally-induced eschars, the hallmark gross lesions consistently found in human cases of *R*. *parkeri* rickettsiosis, [3–10], were reproduced at all cutaneous *R*. *parkeri* inoculation sites in this study. Histologically these lesions were characterized by diffuse infiltrates of macrophages and neutrophils in the acute phase of infection and perivascular dermatitis with infiltrates of predominantly lymphocytes and plasma cells in the chronic phase of infection, both of which have been described in human cases of *R*. *parkeri* rickettsiosis [3–7,10]. Furthermore, similar to human cases of *R*. *parkeri* rickettsiosis, mild to marked dermal vasculitis was noted in two macaques inoculated with *R*. *parkeri* during the acute phase of infection and maculopapular rashes were noted in both macaques in the tick + *R*. *parkeri* group [3–9]. Anti-*Rickettsia* IHC revealed the presence of few to many organisms within cutaneous inoculation sites primarily within macrophages and occasionally neutrophils as identified by nuclear morphology. The presence of *R*. *parkeri* primarily within inflammatory cells within cutaneous lesions as opposed to endothelial cells is similar to what is reported in the literature for human cases of *R*. *parkeri* rickettsiosis [3–6], an interesting finding that requires further study given the predilection of other SFG *Rickettsia* for endothelial cell infection.

Tick feeding during *R*. *parkeri* inoculation consistently resulted in enhanced gross lesions as well as a greater systemic inflammatory response in the acute phase of infection. Interestingly, tick feeding during *R*. *parkeri* inoculation did not have an effect on cutaneous rickettsial load at 4 dpi with decreased numbers of *R*. *parkeri* detected at 9 dpi. This is in contrast to previous studies in mice, where nymphal tick feeding post-*R*. *parkeri* inoculation resulted in increased

bacterial load in the skin at 8 dpi [12]. This difference could be an artifact of sampling, where, despite our best efforts, the 4-mm biopsies may not have been representative of the overall lesion. We also cannot rule out the possibility that tick feeding prior to inoculation primed the immune response leading to increased clearance of bacteria at the inoculation site. Nevertheless, *R. parkeri* DNA and rare organisms were detected in a lymph node of an animal in the tick + *R. parkeri* group by qPCR and IHC, as well as at the site of inoculation at 32 dpi by qPCR. These results suggest that tick feeding may facilitate dissemination and persistence of *R. parkeri*. However, significance of these findings should not be overstated as they are based on data from one animal. Future study with larger animal numbers would be needed to confirm these results.

The presence of rare *Rickettsia* noted in the tick-only animal at 4 dpi in the skin by IHC is attributed to transmission of *Candidatus* "Rickettsia andeanae," a rickettsial species with no known pathogenicity, which has been detected in wild-caught *A. maculatum* [42,61–73] and was detected in low prevalence in the larval progeny of the ticks used in this experiment. The observed transmission of low numbers of *Candidatus* "Rickettsia andeanae" has been previously reported when persistently infected *A. maculatum* nymphs were fed on mice [12]. Similar to this previous report, rare bacteria were noted in the tick-only animal in this study via IHC, but not by either qPCR or two rounds of traditional PCR. This finding could indicate that IHC is more sensitive than PCR in detecting low rickettsial loads after DNA extraction, or it could be a result of sampling error, where low numbers of *Rickettsia* were present in the tissue sample for IHC, but not sampled in the tissue section for PCR. The amount of disease caused by transmission of *Candidatus* "Rickettsia andeanae" in this study is uncertain, as mild peripheral neutrophilia and marked neutrophilic dermatitis were detected at 4 dpi in this animal without elevations of inflammatory cytokines or acute phase proteins in the peripheral blood. This inflammation could be attributed to the tick inoculation of bacteria; however, an inflammatory reaction to the partially purified Vero cell lysate injection or tick feeding could not be ruled out. Ideally, future study would include Vero lysate injection alone, *Candidatus* "Rickettsia andeanae" injection alone, and *Rickettsia*-free tick feeding as additional experimental groups. The lack of anti-*Rickettsia* antibody production in the tick-only animal indicates that the innate immune response alone is likely sufficient to clear the *Candidatus* "Rickettsia andeanae." Further study is needed to characterize the pathogenic potential of this organism in comparison to a known human pathogen like *R. parkeri*. However, such a study would rely upon the *in vitro* propagation of *Candidatus* "Rickettsia andeanae," which has been proven difficult to culture, growing slowly and in low numbers in mammalian, insect and tick cell lines [67,68].

In summary, rhesus macaques prove to be a valuable animal model for studying the immunobiology of *R. parkeri* rickettsiosis. Intradermal inoculation with *R. parkeri* resulted in eschar and rash formation with characteristic dermatitis, dermal vasculitis, and epidermal necrosis that has been well described in human cases of *R. parkeri* rickettsiosis [3,5,6,10]. Tick feeding during *R. parkeri* inoculation led to increased lesion size and a greater acute phase response with increased persistence of the pathogen and inflammation in the chronic phase. Further study to characterize the influence of immunomodulatory factors introduced by tick feeding at the cutaneous interface that potentially enhance *R. parkeri* pathogenicity is required and should be considered when developing therapeutic strategies and vaccine candidates aimed at blocking transmission of SFG rickettsioses.

Acknowledgments

We thank Dr. Daniel Sonenshine (Old Dominion University) for providing the *Rickettsia parkeri*-free *Amblyomma maculatum* ticks used in this study, Dr. Juan Martinez (Louisiana State

University) for providing the RC_{PFA} antibody, Del Philips (Louisiana State University) for her assistance with the immunohistochemical staining, Emma Harris (Louisiana State University) for providing an *R. parkeri* specific qPCR probe, and Jaqueline Macaluso for her review of the manuscript. This work was part of K. Banajee's doctoral dissertation.

Author Contributions

Conceived and designed the experiments: KHB MEE KRM. Performed the experiments: KHB MEE LAD NRH. Analyzed the data: KHB IML. Contributed reagents/materials/analysis tools: KHB MEE IML LAD NRH KRM. Wrote the paper: KHB KRM.

References

1. Dumler JS. Fitness and freezing: vector biology and human health. J Clin Invest. 2010; 120: 3087–3090. doi: 10.1172/JCI44402 PMID: 20739748
2. Adams DA, Jajosky RA, Ajani U, Kriseman J, Sharp P, Onwen DH, et al. Summary of notifiable diseases—United States, 2012. MMWR Morb Mortal Wkly Rep. 2014; 61: 1–121. PMID: 25233134
3. Paddock CD, Sumner JW, Comer JA, Zaki SR, Goldsmith CS, Goddard J, et al. *Rickettsia parkeri*: a newly recognized cause of spotted fever rickettsiosis in the United States. Clin Infect Dis. 2004; 38: 805–811. PMID: 14999622
4. Whitman TJ, Richards AL, Paddock CD, Tamminga CL, Sniezek PJ, Jiang J, et al. *Rickettsia parkeri* infection after tick bite, Virginia. Emerg Infect Dis. 2007; 13: 334–336. PMID: 17479907
5. Paddock CD, Finley RW, Wright CS, Robinson HN, Schrodt BJ, Lane CC, et al. *Rickettsia parkeri* rickettsiosis and its clinical distinction from Rocky Mountain spotted fever. Clin Infect Dis. 2008; 47: 1188–1196. doi: 10.1086/592254 PMID: 18808353
6. Cragun WC, Bartlett BL, Ellis MW, Hoover AZ, Tyring SK, Mendoza N, et al. The expanding spectrum of eschar-associated rickettsioses in the United States. Arch Dermatol. 2010; 146: 641–648. doi: 10.1001/archdermatol.2010.48 PMID: 20404224
7. Romer Y, Seijo AC, Crudo F, Nicholson WL, Varela-Stokes A, Lash RR, et al. *Rickettsia parkeri* rickettsiosis, Argentina. Emerg Infect Dis. 2011; 17: 1169–1173. doi: 10.3201/eid1707.101857 PMID: 21762568
8. Portillo A, Garcia-Garcia C, Sanz MM, Santibanez S, Venzal JM, Oteo JA. A confirmed case of *Rickettsia parkeri* infection in a traveler from Uruguay. Am J Trop Med Hyg. 2013; 89: 1203–1205. doi: 10.4269/ajtmh.13-0436 PMID: 24166040
9. Romer Y, Nava S, Govedic F, Cicuttin G, Denison AM, Singleton J, et al. *Rickettsia parkeri* rickettsiosis in different ecological regions of Argentina and its association with *Amblyomma tigrinum* as a potential vector. Am J Trop Med Hyg. 2014; 91: 1156–1160. doi: 10.4269/ajtmh.14-0334 PMID: 25349376
10. Kaskas NM, Ledet JJ, Wong A, Muzny CA, Elopre L, Hughey L. *Rickettsia parkeri*: eschar diagnosis. J Am Acad Dermatol. 2014; 71: e87–89. doi: 10.1016/j.jaad.2014.03.024 PMID: 25128140
11. Grasperge BJ, Reif KE, Morgan TD, Sunyakumthorn P, Bynog J, Paddock CD, et al. Susceptibility of inbred mice to *Rickettsia parkeri*. Infect Immun. 2012; 80: 1846–1852. doi: 10.1128/IAI.00109-12 PMID: 22392926
12. Grasperge BJ, Morgan TW, Paddock CD, Peterson KE, Macaluso KR. Feeding by *Amblyomma maculatum* (Acari: Ixodidae) enhances *Rickettsia parkeri* (Rickettsiales: Rickettsiaceae) infection in the skin. J Med Entomol. 2014; 51: 855–863. PMID: 25118419
13. Hoshino K, Takeuchi O, Kawai T, Sanjo H, Ogawa T, Takeda Y, et al. Cutting edge: Toll-like receptor 4 (TLR4)-deficient mice are hyporesponsive to lipopolysaccharide: evidence for TLR4 as the LPS gene product. J Immunol. 1999; 162: 3749–3752. PMID: 10201887
14. Jordan JM, Woods ME, Olano J, Walker DH. The absence of Toll-like receptor 4 signaling in C3H/HeJ mice predisposes them to overwhelming rickettsial infection and decreased protective Th1 responses. Infect Immun. 2008; 76: 3717–3724. doi: 10.1128/IAI.00311-08 PMID: 18490467
15. Moraru GM, Goddard J, Paddock CD, Varela-Stokes A. Experimental infection of cotton rats and bobwhite quail with *Rickettsia parkeri*. Parasit Vectors. 2013; 6: 70. doi: 10.1186/1756-3305-6-70 PMID: 23497681
16. La Scola B, Bechah Y, Lepidi H, Raoult D. Prediction of rickettsial skin eschars in humans using an experimental guinea pig model. Microb Pathog. 2009; 47: 128–133. doi: 10.1016/j.micpath.2009.06.004 PMID: 19527779

17. Wikel S. Ticks and tick-borne pathogens at the cutaneous interface: host defenses, tick countermeasures, and a suitable environment for pathogen establishment. Front Microbiol. 2013; 4: 337. doi: 10.3389/fmicb.2013.00337 PMID: 24312085

18. Kubes M, Kocakova P, Slovak M, Slavikova M, Fuchsberger N, Nuttall PA. Heterogeneity in the effect of different *Ixodid* tick species on human natural killer cell activity. Parasite Immunol. 2002; 24: 23–28. PMID: 11856443

19. Hajnicka V, Vancova I, Kocakova P, Slovak M, Gasperik J, Slavikova M, et al. Manipulation of host cytokine network by ticks: a potential gateway for pathogen transmission. Parasitology. 2005; 130: 333–342. PMID: 15796016

20. Vancova I, Slovak M, Hajnicka V, Labuda M, Simo L, Peterkova K, et al. Differential anti-chemokine activity of *Amblyomma variegatum* adult ticks during blood-feeding. Parasite Immunol. 2007; 29: 169–177. PMID: 17371454

21. Peterkova K, Vancova I, Hajnicka V, Slovak M, Simo L, Nuttall PA. Immunomodulatory arsenal of nymphal ticks. Med Vet Entomol. 2008; 22: 167–171. doi: 10.1111/j.1365-2915.2008.00726.x PMID: 18498617

22. Carvalho-Costa TM, Mendes MT, da Silva MV, da Costa TA, Tiburcio MG, Anhe AC, et al. Immunosuppressive effects of *Amblyomma cajennense* tick saliva on murine bone marrow-derived dendritic cells. Parasit Vectors. 2015; 8: 22. doi: 10.1186/s13071-015-0634-7 PMID: 25586117

23. Nuttall PA, Labuda M. Tick–host interactions: saliva-activated transmission. Parasitology. 2004; 129: S177–S189. PMID: 15938511

24. Troughton DR, Levin ML. Life cycles of seven ixodid tick species (Acari: Ixodidae) under standardized laboratory conditions. J Med Entomol. 2007; 44: 732–740. PMID: 17915502

25. Regnery RL, Spruill CL, Plikaytis BD. Genotypic identification of rickettsiae and estimation of intraspecies sequence divergence for portions of two rickettsial genes. J Bacteriol. 1991; 173: 1576–1589. PMID: 1671856

26. Fournier PE, Roux V, Raoult D. Phylogenetic analysis of spotted fever group rickettsiae by study of the outer surface protein rOmpA. Int J Syst Bacteriol. 1998; 48 Pt 3: 839–849.

27. Pornwiroon W, Pourciau SS, Foil LD, Macaluso KR. *Rickettsia felis* from cat fleas: isolation and culture in a tick-derived cell line. Appl Environ Microbiol. 2006; 72: 5589–5595. PMID: 16885313

28. Weiss E. Growth and physiology of rickettsiae. Bacteriol Rev. 1973; 37: 259–283. PMID: 4585793

29. Petchampai N, Sunyakumthorn P, Guillotte ML, Thepparit C, Kearney MT, Mulenga A, et al. Molecular and functional characterization of vacuolar-ATPase from the American dog tick *Dermacentor variabilis*. Insect Mol Biol. 2014; 23: 42–51. doi: 10.1111/imb.12059 PMID: 24164319

30. Kurtti TJ, Simser JA, Baldridge GD, Palmer AT, Munderloh UG. Factors influencing *in vitro* infectivity and growth of *Rickettsia peacockii* (Rickettsiales: Rickettsiaceae), an endosymbiont of the Rocky Mountain wood tick, *Dermacentor andersoni* (Acari, Ixodidae). J Invertebr Pathol. 2005; 90: 177–186. PMID: 16288906

31. Feng HM, Whitworth T, Olano JP, Popov VL, Walker DH. Fc-dependent polyclonal antibodies and antibodies to outer membrane proteins A and B, but not to lipopolysaccharide, protect SCID mice against fatal *Rickettsia conorii* infection. Infect Immun. 2004; 72: 2222–2228. PMID: 15039346

32. Monje LD, Nava S, Antoniazzi LR, Colombo VC, Beldomenico PM. In vitro isolation and infection intensity of *Rickettsia parkeri* in *Amblyomma triste* ticks from the Parana River Delta region, Argentina. Ticks Tick Borne Dis. 2014; 5: 924–927. doi: 10.1016/j.ttbdis.2014.07.002 PMID: 25113981

33. Sammons LS, Kenyon RH, Hickman RL, Pedersen CE, Jr. Susceptibility of laboratory animals to infection by spotted fever group rickettsiae. Lab Anim Sci. 1977; 27: 229–234. PMID: 857087

34. Feng HM, Wen J, Walker DH. *Rickettsia australis* infection: a murine model of a highly invasive vasculopathic rickettsiosis. Am J Pathol. 1993; 142: 1471–1482. PMID: 8494048

35. Eremeeva ME, Liang Z, Paddock C, Zaki S, Vandenbergh JG, Dasch GA, et al. *Rickettsia rickettsii* infection in the pine vole, *Microtus pinetorum*: kinetics of infection and quantitation of antioxidant enzyme gene expression by RT-PCR. Ann N Y Acad Sci. 2003; 990: 468–473. PMID: 12860675

36. Bechah Y, Capo C, Grau GE, Raoult D, Mege JL. A murine model of infection with *Rickettsia prowazekii*: implications for pathogenesis of epidemic typhus. Microbes Infect. 2007; 9: 898–906. PMID: 17537665

37. Horta MC, Sabatini GS, Moraes-Filho J, Ogrzewalska M, Canal RB, Pacheco RC, et al. Experimental infection of the opossum *Didelphis aurita* by *Rickettsia felis*, *Rickettsia bellii*, and *Rickettsia parkeri* and evaluation of the transmission of the infection to ticks *Amblyomma cajennense* and *Amblyomma dubitatum*. Vector Borne Zoonotic Dis. 2010; 10: 959–967. doi: 10.1089/vbz.2009.0149 PMID: 20455783

38. Embers ME, Grasperge BJ, Jacobs MB, Philipp MT. Feeding of ticks on animals for transmission and xenodiagnosis in Lyme disease research. J Vis Exp. 2013.

39. Sonenshine DE. Pheromones and other semiochemicals of ticks and their use in tick control. Parasitology. 2004; 129: S405–S425. PMID: 15938521
40. Graf PC, Chretien JP, Ung L, Gaydos JC, Richards AL. Prevalence of seropositivity to spotted fever group rickettsiae and *Anaplasma phagocytophilum* in a large, demographically diverse US sample. Clin Infect Dis. 2008; 46: 70–77. doi: 10.1086/524018 PMID: 18171216
41. Chan YG, Riley SP, Chen E, Martinez JJ. Molecular basis of immunity to rickettsial infection conferred through outer membrane protein B. Infect Immun. 2011; 79: 2303–2313. doi: 10.1128/IAI.01324-10 PMID: 21444665
42. Wright CL, Nadolny RM, Jiang J, Richards AL, Sonenshine DE, Gaff HD, et al. *Rickettsia parkeri* in gulf coast ticks, southeastern Virginia, USA. Emerg Infect Dis. 2011; 17: 896–898. doi: 10.3201/eid1705.101836 PMID: 21529406
43. Bruce AG, Bakke AM, Thouless ME, Rose TM. Development of a real-time QPCR assay for the detection of RV2 lineage-specific rhadinoviruses in macaques and baboons. Virol J. 2005; 2: 2. PMID: 15634356
44. Reif KE, Kearney MT, Foil LD, Macaluso KR. Acquisition of *Rickettsia felis* by cat fleas during feeding. Vector Borne Zoonotic Dis. 2011; 11: 963–968. doi: 10.1089/vbz.2010.0137 PMID: 21214386
45. Cray C, Zaias J, Altman NH. Acute phase response in animals: a review. Comp Med. 2009; 59: 517–526. PMID: 20034426
46. Murphy KP. The induced responses of innate immunity. In: Murphy KP, editor. Janeway's Immunobiology. 8th ed. New York: Garland Science; 2012. p. 75–125.
47. Ceron JJ, Eckersall PD, Martýnez-Subiela S. Acute phase proteins in dogs and cats: current knowledge and future perspectives. Vet Clin Pathol. 2005; 34: 85–99. PMID: 15902658
48. Stockham SL, Scott MA. Leukocytes. In: Stockham SL, Scott MA, editors. Fundamentals of Veterinary Clinical Pathology. 2nd ed. Ames, IA: Blackwell Publishing Ltd; 2008. p. 53–106.
49. Imhof BA, Dunon D. Leukocyte migration and adhesion. Adv Immunol. 1995; 58: 345–516. PMID: 7537936
50. Vitale G, Mansueto S, Gambino G, Mocciaro C, Spinelli A, Rini GB, et al. The acute phase response in Sicilian patients with boutonneuse fever admitted to hospitals in Palermo, 1992–1997. J Infect. 2001; 42: 33–39. PMID: 11243751
51. Walker DH, Ismail N. Emerging and re-emerging rickettsioses: endothelial cell infection and early disease events. Nat Rev Microbiol. 2008; 6: 375–386. doi: 10.1038/nrmicro1866 PMID: 18414502
52. Brossard M, Wikel S. Tick immunobiology. In: Bowman AS, Nuttall P. A., editor. Ticks: biology, disease and control. Cambridge: Cambridge University Press; 2008. p. 186–204.
53. Schoeler GB, Wikel SK. Modulation of host immunity by haematophagous arthropods. Ann Trop Med Parasitol. 2001; 95: 755–771. PMID: 11784430
54. Kazimirova M, Stibraniova I. Tick salivary compounds: their role in modulation of host defences and pathogen transmission. Front Cell Infect Microbiol. 2013; 3: 43. doi: 10.3389/fcimb.2013.00043 PMID: 23971008
55. Schoeler GB, Manweiler SA, Wikel SK. *Ixodes scapularis*: effects of repeated infestations with pathogen-free nymphs on macrophage and T lymphocyte cytokine responses of BALB/c and C3H/HeN mice. Exp Parasitol. 1999; 92: 239–248. PMID: 10425152
56. Schoeler GB, Manweiler SA, Wikel SK. Cytokine responses of C3H/HeN mice infested with *Ixodes scapularis* or *Ixodes pacificus* nymphs. Parasite Immunol. 2000; 22: 31–40. PMID: 10607289
57. Locksley RM, Heinzel FP, Sadick MD, Holaday BJ, Gardner KD Jr. Murine cutaneous leishmaniasis: susceptibility correlates with differential expansion of helper T-cell subsets. Ann Inst Pasteur Immunol. 1987; 138: 744–749. PMID: 2964247
58. Reiner SL, Locksley RM. The regulation of immunity to *Leishmania major*. Annu Rev Immunol. 1995; 13: 151–177. PMID: 7612219
59. Muller I, Pedrazzini T, Farrell JP, Louis J. T-cell responses and immunity to experimental infection with *Leishmania major*. Annu Rev Immunol. 1989; 7: 561–578. PMID: 2653376
60. Lopez JE, Vinet-Oliphant H, Wilder HK, Brooks CP, Grasperge BJ, Morgan TW, et al. Real-Time monitoring of disease progression in rhesus macaques infected with *Borrelia turicatae* by tick bite. J Infect Dis. 2014; 210: 1639–1648. doi: 10.1093/infdis/jiu306 PMID: 24879799
61. Blair PJ, Jiang J, Schoeler GB, Moron C, Anaya E, Cespedes M, et al. Characterization of spotted fever group rickettsiae in flea and tick specimens from northern Peru. J Clin Microbiol. 2004; 42: 4961–4967. PMID: 15528680

62. Leydet BF Jr., Liang FT. Detection of human bacterial pathogens in ticks collected from Louisiana black bears (*Ursus americanus luteolus*). Ticks Tick Borne Dis. 2013; 4: 191–196. doi: 10.1016/j.ttbdis.2012.12.002 PMID: 23415850
63. Jiang J, Stromdahl EY, Richards AL. Detection of *Rickettsia parkeri* and *Candidatus* Rickettsia andeanae in *Amblyomma maculatum* Gulf Coast ticks collected from humans in the United States. Vector Borne Zoonotic Dis. 2012; 12: 175–182. doi: 10.1089/vbz.2011.0614 PMID: 22022815
64. Flores-Mendoza C, Florin D, Felices V, Pozo EJ, Graf PC, Burrus RG, et al. Detection of *Rickettsia parkeri* from within Piura, Peru, and the first reported presence of *Candidatus* Rickettsia andeanae in the tick *Rhipicephalus sanguineus*. Vector Borne Zoonotic Dis. 2013; 13: 505–508. doi: 10.1089/vbz.2012.1028 PMID: 23488453
65. Paddock CD, Denison AM, Dryden MW, Noden BH, Lash RR, Abdelghani SS, et al. High prevalence of "*Candidatus* Rickettsia andeanae" and apparent exclusion of *Rickettsia parkeri* in adult *Amblyomma maculatum* (Acari: Ixodidae) from Kansas and Oklahoma. Ticks Tick Borne Dis. 2015;
66. Fornadel CM, Zhang X, Smith JD, Paddock CD, Arias JR, Norris DE. High rates of *Rickettsia parkeri* infection in Gulf Coast ticks (*Amblyomma maculatum*) and identification of "*Candidatus* Rickettsia andeanae" from Fairfax County, Virginia. Vector Borne Zoonotic Dis. 2011; 11: 1535–1539. doi: 10.1089/vbz.2011.0654 PMID: 21867421
67. Luce-Fedrow A, Wright C, Gaff HD, Sonenshine DE, Hynes WL, Richards AL. *In vitro* propagation of *Candidatus* Rickettsia andeanae isolated from *Amblyomma maculatum*. FEMS Immunol Med Microbiol. 2012; 64: 74–81. doi: 10.1111/j.1574-695X.2011.00905.x PMID: 22098316
68. Ferrari FAG, Goddard J, Moraru GM, Smith WEC, Varela-Stokes AS. Isolation of "*Candidatus* Rickettsia andeanae" (Rickettsiales: Rickettsiaceae) in embryonic cells of naturally infected *Amblyomma maculatum* (Ixodida: Ixodidae). J Med Entomol. 2013; 50: 1118–1125. PMID: 24180118
69. Paddock CD, Fournier PE, Sumner JW, Goddard J, Elshenawy Y, Metcalfe MG, et al. Isolation of *Rickettsia parkeri* and identification of a novel spotted fever group *Rickettsia* sp. from Gulf Coast ticks (*Amblyomma maculatum*) in the United States. Appl Environ Microbiol. 2010; 76: 2689–2696. doi: 10.1128/AEM.02737-09 PMID: 20208020
70. Jiang J, Blair PJ, Felices V, Moron C, Cespedes M, Anaya E, et al. Phylogenetic analysis of a novel molecular isolate of spotted fever group Rickettsiae from northern Peru: Candidatus Rickettsia andeanae. AnnNYAcadSci. 2005; 1063: 337–342.
71. Ferrari FA, Goddard J, Paddock CD, Varela-Stokes AS. *Rickettsia parkeri* and *Candidatus* Rickettsia andeanae in Gulf Coast ticks, Mississippi, USA. Emerg Infect Dis. 2012; 18: 1705–1707. doi: 10.3201/eid1810.120250 PMID: 23018026
72. Varela-Stokes AS, Paddock CD, Engber B, Toliver M. *Rickettsia parkeri* in *Amblyomma maculatum* ticks, North Carolina, USA, 2009–2010. Emerg Infect Dis. 2011; 17: 2350–2353. doi: 10.3201/eid1712.110789 PMID: 22172164
73. Nadolny RM, Wright CL, Sonenshine DE, Hynes WL, Gaff HD. Ticks and spotted fever group rickettsiae of southeastern Virginia. Ticks Tick Borne Dis. 2014; 5: 53–57. doi: 10.1016/j.ttbdis.2013.09.001 PMID: 24201057
